# A New Player in Depression: MiRNAs as Modulators of Altered Synaptic Plasticity

**DOI:** 10.3390/ijms23094555

**Published:** 2022-04-20

**Authors:** Ya-Nan Gao, Yong-Qian Zhang, Hao Wang, Yu-Lin Deng, Nuo-Min Li

**Affiliations:** 1 School of Medical Technology, Beijing Institute of Technology, Beijing 100081, China; 3120201946@bit.edu.cn (Y.-N.G.); 3120211996@bit.edu.cn (H.W.); 2 School of Life Science, Beijing Institute of Technology, Beijing 100081, China; zyq@bit.edu.cn (Y.-Q.Z.); deng@bit.edu.cn (Y.-L.D.)

**Keywords:** depression, microRNA, neuroplasticity, synaptic functional plasticity, synaptic structural plasticity

## Abstract

Depression is a psychiatric disorder that presents with a persistent depressed mood as the main clinical feature and is accompanied by cognitive impairment. Changes in neuroplasticity and neurogenesis greatly affect depression. Without genetic changes, epigenetic mechanisms have been shown to function by regulating gene expression during the body’s adaptation to stress. Studies in recent years have shown that as important regulatory factors in epigenetic mechanisms, microRNAs (miRNAs) play important roles in the development and progression of depression through the regulation of protein expression. Herein, we review the mechanisms of miRNA-mediated neuroplasticity in depression and discus synaptic structural plasticity, synaptic functional plasticity, and neurogenesis. Furthermore, we found that miRNAs regulate neuroplasticity through several signalling pathways to affect cognitive functions. However, these pathways do not work independently. Therefore, we try to identify synergistic correlations between miRNAs and multiple signalling pathways to broaden the potential pathogenesis of depression. In addition, in the future, dual-function miRNAs (protection/injury) are promising candidate biomarkers for the diagnosis of depression, and their regulated genes can potentially be used as target genes for the treatment of depression.

## 1. Introduction

Depression (Major Depressive Disorder, MDD) is one of the most prevalent psychiatric disorders, which has a multifactorial origin including genetic and environmental factors [1,2]. The clinical presentation of the patient is dominated by a prolonged mood of sadness or irritability, along with symptoms that significantly affect cognitive and neuroenergetic aspects such as difficulty concentrating, delayed response, memory changes, anorexia, and sleep disturbances [3]. According to the World Health Organization (WHO), the number of individuals suffering from depression worldwide will reach 322 million in 2025. The total number of individuals with depression increased 18.4% from 2005 to 2015, with the percentage of affected individuals increasing year-to-year; therefore, depression is listed by the WHO as the top-ranked disease in the world [4,5]. Imaging studies indicate that the brain areas associated with mood and cognition shrink in people with depression [6,7]. This shrinkage is closely associated with neuroplasticity, which includes nerve damage, changes in synaptic function and structure, and even damage of the synaptic circuits [8]. For instance, the microglia are activated abnormally, the number of mature oligodendrocytes is reduced [9], and the prefrontal cortex activity is decreased [10]. In the hippocampus, the density of dendritic spines is reduced [11], accompanied by the mitochondrial dysfunction and a decreased number of synaptic vesicles [12]. Currently, there are a few drugs available to relieve mood disorders in people with depression; however, they are not effective for the cognitive dysfunction in depression. In addition, there is a lack of effective markers for the diagnoses of depression, which rely on individual subjective responses and the nature of the heterogeneous disorder [13,14]. Fortunately, the content and types of microRNAs (miRNAs) in the human brain are more abundant than those in the brain of other primates, especially in the cognition-associated prefrontal cortex [15]. In nervous systems, miRNAs affect the development and functions of neurons and participate in the development and progression of various neurodegenerative diseases, including Alzheimer’s disease, Parkinson’s disease, Huntington’s disease, and depression [16]. However, the mechanism underlying the regulation of synaptic plasticity by miRNAs in depression is still unclear. Herein, we discuss the possible mechanisms of miRNAs in the regulation of synaptic plasticity in depression and try to identify the synergistic correlations between miRNAs and multiple signalling pathways to confirm their potential contributions in the management of depression in the future.

## 2. MiRNAs and Neuroplasticity in Depression

**Figure 1:**
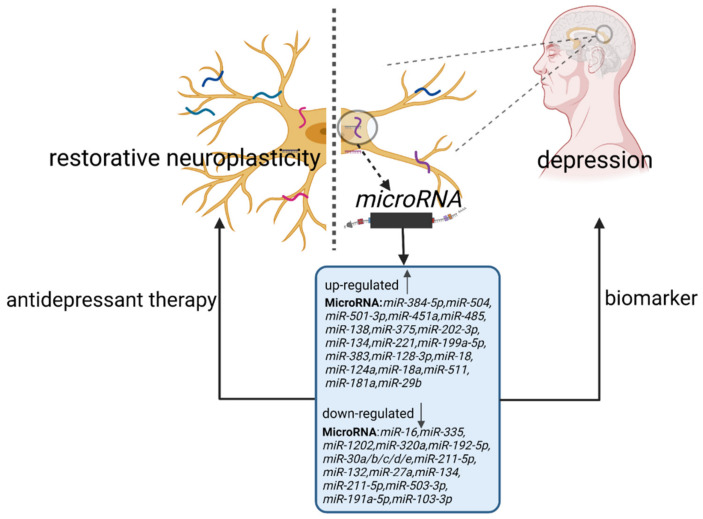
The involvement of miRNAs in the regulation of neuroplasticity in patients with depression. The up or downregulation of miRNA expression levels leads to impaired neuroplasticity and is involved in the pathogenesis of depression. MiRNAs can not only serve as biomarkers for diagnosing depression, but they also function as targets for antidepressant drugs to modulate neuroplasticity and improve depressive symptoms.

Neuroplasticity, also known as neural plasticity or brain plasticity, is a process that involves adaptive morphology, connectivity, and functions of neuronal network changes in response to internal or external stimuli [17]. Neuroplasticity is closely associated with the development of depression. MiRNAs are involved in this process and play important roles [18] (Figure 1).

### 2.1. Biogenesis and Function of MiRNAs

MiRNAs are the class of small noncoding single-strand RNAs with a length of 18–25 nucleotides that are involved in the regulation of post-transcriptional gene expression. The biogenesis of miRNAs has been well described. In general, RNA polymerase II transcribed the DNA to produce a primary miRNA (pri-miRNA) in the nucleus, which is cleaved by ribozyme III Drosha, cofactor DGCR8, and its cofactor TRBP, to become precursor miRNA (pre-miRNA). Pre-miRNAs are delivered to the cytoplasm by transporter exportin−5 where they are cleaved into mature miRNAs by cytoplasmic Rnase III Dicer. They inhibit gene expression levels by binding to 3′ untranslated region (3′UTR) of mRNA, leading to mRNA degradation or translation inhibition [19,20]. One single miRNA can regulate multiple target genes, and one single target gene can also be regulated by multiple miRNAs, indicating that the expression of multiple proteins in cells may act synergistically. Many miRNAs are expressed in large quantities in the nervous system in a tissue-specific manner, for example, in axons, dendritic spines, and presynaptic membranes to participate in the development and progression of neuroplasticity, including neurogenesis, neuronal maturation, synaptic formation, axon guidance, and neurite growth [21].

### 2.2. MiRNAs and Synaptic Plasticity

**Figure 2:**
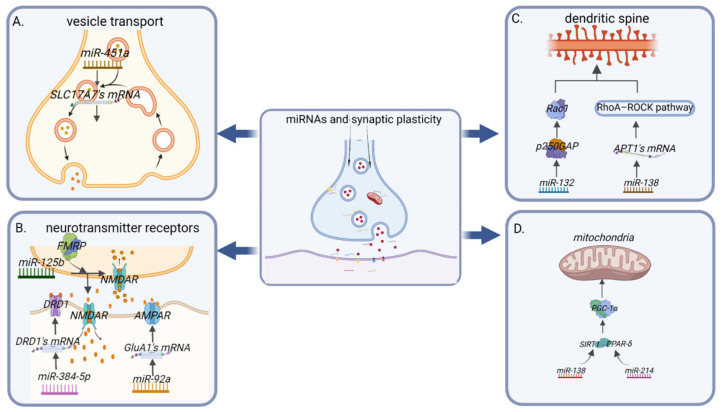
MiRNAs regulate synaptic plasticity. (**A**) In terms of neurotransmitter transmission, on the one hand, miRNAs regulate vesicle transport; e.g., miR-451a can target the glutamate transporter protein (SLC17A7) and regulate neurotransmitter transmission. (**B**) On the other hand, miRNAs regulate the density and activity of neurotransmitter-related receptors on the presynaptic and postsynaptic membranes; e.g., miR-384-5P directly targets dopamine D1-like receptors (DRD1) and affects the expression levels of dopamine (DA). MiR-92a directly targets inhibition of translation of the AMPA receptor subunit GluA1, while miR-125b and fragile X mental retardation protein (FMRP) affect NMDA receptor activity by inhibiting Grin2a gene expression, and ultimately both miR-92a and miR-125b affect glutamate expression levels. (**C**) MiRNAs directly affect synaptic dendritic spine formation-related proteins; e.g., miR-132 inhibits the activity of GTPase-activating protein (p250GAP) and increases spine onset. However, miR-138 inhibits translation of acyl protein thioesterase 1 (APT1) mRNA, which in turn activates the downstream RhoA–ROCK pathway, leading to small spines. (**D**) MiRNAs affect mitochondrial function in synapses; e.g., miR-138 and miR-214 both indirectly affect the activity of the peroxisome proliferator-activated receptor gamma-coactivator-1α (PGC-1α), affecting mitochondrial biogenesis and redox reactions.

MRNAs and their translated proteins in neuronal dendrites and axons promote synaptic production and its maintenance. Mature miRNAs and pre-miRNAs localised in neurons participate in the regulation of synaptic protein synthesis [22]. MiRNAs have different distributions in subcellular regions in a manner similar to their distribution of axonal mRNA expression. When localised stimulation or injury occurs, specific miRNAs are transported to axons in forms of pre-miRNA. The levels of these pre-miRNAs change with injuries to regulate the translation of local mRNAs in axons [23]. Changes in synaptic structures and functions are called synaptic plasticity. Structural and functional plasticity are two interlinked, mutually-depended forms of synaptic plasticity, and both are associated with transmission efficiency and memory storage [24]. (Figure 2).

#### 2.2.1. MiRNAs and Synaptic Functional Plasticity

Depending on the activity of neighbouring network and external stimuli, synapses that are functioning can be selectively modified to strengthen or weaken synaptic connections and change synaptic functions. MiRNAs can participate in the regulation of synaptic functions by influencing some protein factors [25].

##### MiRNAs and Neurotransmission-Related Receptors

The release of neurotransmitters plays a very important role in synaptic plasticity and learning and memory. The efficiency of synaptic transmission is closely associated with the density and activity of neurotransmitter receptors and the formation, assembly, and transport of synaptic vesicles. Some miRNAs affect the response to neurotransmitters by altering the density and function of receptors and their reuptake by changing the function of transporters (Table 1). Serotonin transporter (SERT) is a monoamine transporter protein. It reduces the concentration of 5-hydroxytryptamine (5-HT) in the extracellular fluid of nerve cells to regulate the neurotransmission of 5-HT [26]. MiR-16 targets the 3′UTR of *SERT* mRNA to regulate its translation [27,28]. In the cerebrospinal fluid (CSF) of patients with major depressive disorder, the expression levels of miR-16 and 5-HT were significantly downregulated. In addition, the level of reduction negatively correlated with depression severity [29]. However, opposite results were obtained in a rat model of chronic unpredictable mild stress (CUMS) [30]. D1-like receptor (DRD1) was a potential target regulated by miR-384-5p. The inhibition or overexpression of miR-384-5p in the prefrontal cortex in a rat model of attention deficit hyperactivity disorder affected DRD1 gene expression and changed the protein level of the dopamine transporter (DAT) [31,32]. Changes in DAT expression affected the reuptake of DA by the presynaptic membrane, changed the DA level, and regulated the learning and memory level of rats [32]. Furthermore, miR-504 in the nucleus accumbens in a rat model of CUMS was shown to target and regulate the DRD1 level [33] similar to the function of miR-384-5p. Therefore, the upregulation of miR-384-5p and miR-504 inhibited DR expression and affected the DA concentration in the synaptic cleft to influence the development of depression.

Glutamate is involved in multiple signalling pathways through the N-methyl-D-aspartate (NMDA) receptors. Excessive release of glutamate in depression can cause neuronal toxicity and lead to neuronal death. NR2A (*Grin2a*) is a subunit of the ionotropic glutamate receptor NMDA receptor, which is significantly upregulated in the brain of patients with depression and suicide [34]. MiR-125b and fragile X mental retardation protein (FMRP) were shown to affect the activity of the NMDA receptor and glutamate transmission through the inhibition of *Grin2a* gene expression [35]. Furthermore, some studies showed that miR-320a in the plasma of patients with depression targeted and regulated Grin2a [36], whereas a reduction in *Grin2a* levels caused a significant reduction in long-term potentiation (LTP) and impaired spatial learning and memory [37,38]. A study by Letellier et al. found that miR-92a in hippocampal neurons selectively bound to the 3′UTR of *GluA1*, a subunit of the α-amino-3-hydroxy-5-methyl-4-isoxazolepropionic acid (AMPA) receptor, to inhibit its translation [39]. It has also been shown that miR-137 [40,41] and miR-501-3p [42,43] target *GluA1* to regulate AMPA receptor-mediated synaptic currents and the number of functional synapses. Morquette et al. found that miR-223 targeted the 3′UTR of *GluA2* and *NR2B* mRNAs to reduce the levels of GluA2 and NR2B, inhibit NMDA-induced Ca^2+^ influx in hippocampal neurons, and protect the brain from injury caused by Glu-induced neuronal hyperexcitation [44,45]. When miR-223 in the hippocampus of mice was knocked out, the NR2B and GluA2 levels significantly increased, promoting an NMDA-induced Ca^2+^ influx, inducing miniature excitatory postsynaptic currents (mEPSC) from hippocampal neurons and prolonging the potential recovery time of glutamatergic neurotransmitters [46]. In patients with depression, glutamate metabotropic receptor 4 (GRM4) expression increased [47,48,49], and miR-335 was significantly downregulated [50]. Antidepressant drug treatment with citalopram upregulated miR-335 expression and inhibited GRM4 expression [51]. Studies showed that miR-335 targeted the 3′UTR of *GRM4* mRNA to regulate its expression. In addition, GRM4 negatively regulated miR-335 expression [51]. There was a check and balance relationship between these two. Some studies also showed that miR-1202 had functions similar to those of miR-335 and targeted GRM4 to negatively regulate its expression level [52]. Treatment with citalopram or imipramine significantly suppressed GRM4 expression [53] and regulated the glutamatergic system to achieve antidepression functions. In addition, perisynaptic astrocytes expressed excitatory amino acid transporter 2 (EAAT2, rodent analogue GLT1) [54], which regulates the concentration of glutamate in the synaptic cleft and extrasynaptic glutamate [55]. The specific antisense against miR-124a significantly reduced GLT1 protein expression and glutamate uptake levels in striatum of adult mice without decreasing GLT1 mRNA levels [55]. The results of this study indicated that miRNAs act as a bridge and mediate neuron-to-astrocyte communication in the progression of psychiatric disorders. It provides a new idea for finding therapeutic targets for depression.

##### MiRNAs and Synaptic Vesicle Transport

Some miRNAs can affect synaptic vesicle transport and block the release of neurotransmitters (Table 2). Postmortem autopsy reports of patients with depression showed that vesicular glutamate transporter 1 (Vglut1) mRNA expression was downregulated in the entorhinal cortex [56]. The same result was also obtained in a CUMS model [57]. Glutamate must be packaged into vesicles by vGLuT1 to be later released into the synaptic cleft [58]. MiR-451a targets Vglut1 to negatively regulate its level [36]. Treatment with fluoxetine and ketamine effectively reduced miR-451 levels and regulated Vglut1 expression in patients with depression. MiRNA-15b-5p might reduce excitatory synaptic transmission, innervation, and activity through a reduction in syntaxin-binding protein 3 (STXBP3A)/vesicle-associated protein 1 (VAMP1) expression in the nucleus accumbens to play a critical role in CUMS-induced depression [59]. An analysis was performed by a genome-wide association study (GWAS) to identify neuroticism-, anxiety-, and depression-associated target genes from postmortem human brain tissues. The results indicated that miR-133a and miR-128 in the amygdala generated the same expression pattern and translation inhibition function with regard to synaptic vesicle glycoprotein 2A (SV2A) regulation. Cell experiments also confirmed that miR-133a and miR-128 targeted Sv2A 3′UTR to inhibit its expression [60]. Another study on the LPS-induced model showed that miR-96 was targeted and negatively regulated SV2C expression in the hippocampal CA1 region, decreased 5-hydroxyindole acetic acid (5-HT) and dopamine (DA) release, and induced the development of depression [61]. MiR-34a-5p expression increased in the hippocampus of mice exposed to chronic stress. Through the TargetScan database and in vitro experiments, synaptotagmin-1 was confirmed to be a potential binding target of miR-34a-5p. As a major protein in the synaptic vesicle membrane, synaptotagmin-1 may serve as a calcium sensor to regulate neurotransmitter release. After antidepressant drug treatment with fluoxetine, miR-34a-5p was significantly downregulated. Depressive symptoms were relieved, while synaptic plasticity improved through the regulation of synaptotagmin-1 expression [62].

#### 2.2.2. MiRNAs and Synaptic Structural Plasticity

MiRNAs can affect the expression of synapse-associated proteins and active regulatory factors, which consequently influence the morphology and quantity of synaptic structures (dendrites and spines), for example, transformations from a mature mushroom-like morphology to an immature stubby or slender morphology, a reduction in the number of dendrite branches, and regulation of synaptic structural plasticity [63]. Zhang et al. confirmed that miR-132 targeted and inhibited the expression of the GTPase-activating protein (p250GAP). p250GAP knockout increased spine formation, whereas a reduction in miR-132 reduced mEPSC frequency and the number of GluR1-positive spines [64,65]. This effect on spines was achieved through the synapse-specific Kalirin 7-Rac1 signalling pathway. MiR-138 in rat hippocampal neurons inhibited adenine phosphoribosyl transferase 1 (APT1) mRNA translation. APT1 reduction directly increased the palmitoylation of the Ga13 subunit of G protein to further activate the downstream RhoA–ROCK pathway and suppress spine enlargement [66]. MiR-9 in mouse brain targeted the transcription repressor RE1-silencing transcription factor (REST) to promote normal dendrite growth [67,68]. In miR-137 knockout mouse cortex, the upregulation of the histone-lysine N-methyltransferase enzyme enhancer of zeste homolog 2 (EZH2) significantly improved defects caused by the loss of miR-137 and restored dendritic complexity, dendritic length, and the number of dendritic branches [69]. MiR-188 targeted semaphorin 3F receptor (neuropilin-2, Nrp-2) to regulate the number of dendritic spines and mEPSC frequency in hippocampal neurons [70]. Furthermore, miR-485 was shown to be significantly upregulated in the dorsal hippocampus of CUMS mice [71]. Further, a proteomic analysis revealed that miR-485 was associated with proteins such as synuclein α (SNCA), microtubule-associated protein Tau (MAPT), and the NR1 subunit of the NMDA receptor (GRIN1) [71]. Therefore, it has been speculated that miR-485 is closely associated with the above synaptic structure-related proteins and plays a critical role in synaptic structural plasticity. Furthermore, fibroblast growth factor-2 (FGF2) promotes proliferation of neural progenitor cells, enhances synaptic plasticity and axonal branching, and ameliorates cognitive deficit in neurodegenerative diseases and depression [72]. Woodbury et al. found that microglia cells were hyperactivated in CUMS rats, and miR-497 aggravated hippocampal microglia activation by targeting FGF2, suggesting that miR-497 might represent a potential target for the treatment of depression [73].

Calcium homeostasis is very important for synaptic plasticity. MiRNAs can regulate the morphology of dendritic spines through the regulation of Ca homeostasis-related proteins in synapses [74]. Tropomyosin-related receptor kinase type B-T1 (TrkB-T1) was highly expressed in astrocytes and regulated brain-derived neurotrophic factor (BDNF)-induced Ca^2+^ transients [75] to participate in the regulation of dendritic spine morphology. It has been shown that miR-185 regulated TrkB-T1 in the brain of patients with major depressive disorder [75,76], and the TrkB-T1 expression negatively correlated with miR-185. Therefore, it was speculated that miR-185 regulated the Ca^2+^ concentration in and the morphology of dendritic spines by targeting TrKB-T1. Downregulated miR-192-5p in a CUMS mouse model target the 3′UTR of *fibulin-2* (Fbln2) to inhibit expression [77]. MiR-192-5p upregulation or Fbln2 silencing elevated cAMP, BDNF, NR2B, and CaMKII expression and increased dendritic length, the number of branches, and the density of dendritic spines in mouse neurons in the hippocampal dentate gyrus (DG) region. The results of a study with mice with functional loss of the NMDA receptor indicated that miR-191 reduction negatively regulated tropomodulin 2 (Tmod2) expression and promoted Tmod2 expression to reduce the density of dendritic spines [78]. In postmenopausal depression-like mice, miR-99a targeted the 3′UTR of *FKBP51* to negatively regulate its expression. FKBP51 influenced the cytoplasmic localization of the progesterone receptor (PR), and the cytoplasmic localization of PR was very important for its function [79]. Furthermore, studies also showed that microtubules that provided a structural framework for synaptic membrane structure and synaptic vesicles specifically bound to PR to regulate synaptic vesicle formation. Therefore, miR-99a might influence PR localization through the regulation of FKBP51 to regulate synaptic structural plasticity [80] (Table 3).

#### 2.2.3. MiRNAs and Mitochondrial Function

Mitochondria play a critical role in energy production through the metabolism of lipids, steroids, and proteins. They also maintain cellular stability through the regulation of Ca^2+^, reactive oxygen species (ROS), and apoptosis [81]. Therefore, the dysfunction of mitochondria directly affects normal energy metabolism in the body and is closely associated with the pathogenesis of depression. Zou et al. studied CUMS mouse brain tissues and reported that abnormal increases in NADPH oxidase 1 (Nox1) expression were closely associated with miR-298-5p. Nox1 generated superoxide, which is a type of ROS, then reduced the ATP content. Geniposide treatment increased the miR-298-5p concentration, inhibiting NOX1 expression and thus increasing the ATP content, reducing the ROS level, and relieving depressive symptoms [82]. Hypoxia stress in hippocampal neurons caused the abnormal upregulation of miR-210, which targeted and regulated hypoxia-inducible factor-1α (Hif-1α). MiR-210 knockout in neurons promoted ROS levels and increased the density of dendrites in hippocampal neurons through the regulation of Hif-1α [83]. These results indicated that miR-298-5p and miR-210 affected mitochondrial functions by influencing ROS levels. The process of neuronal development requires an increase in mitochondrial genomes and mitochondrial proteins to promote mitochondrial biogenesis [81]. In a CUMS mouse model, miR-138 upregulation was associated with reduced NAD-dependent deacetylase sirtuin-1 (SIRT1) [84,85] expression. SIRT1 directly interacted with peroxisome proliferator-activated receptor-γ coactivator-1α (PGC-1α). PGC-1α plays important roles in mitochondrial biogenesis and redox reaction pathways [86] and participates in the formation and maintenance of neuronal dendritic spines [87]. Fibronectin type III domain containing 5 (FNDC5) is a PGC-1α-dependent actin. FNDC5 deficiency affects the development of neuronal precursors into mature neurons [84]. Therefore, the regulation of miR-138 concentration may regulate mitochondrial biogenesis through the Sirt1/PGC-1α/FNDC5 pathway to further promote the axonal growth of cortical neurons and neuronal synaptic plasticity. Furthermore, studies showed that miR-214 in the medial prefrontal cortex of mice exhibiting pleasure deficits and failed social interactions in the CSDS mice model targets peroxisome proliferator-activated receptor-δ (PPAR-δ) to improve depression-like behaviour in mice [88]. PPAR-δ also directly regulated PGC-1α expression [89]. MiR-214 inhibition increased the amplitude of mEPSC and the number of dendritic spines in the prefrontal cortex by targeting PPAR- δ to improve depression-like behaviour in CSDS mice [90]. Some studies showed that Twist family BHLH transcription factor 1 (TWIST1) in the prefrontal cortex was a key molecule that mediated depression-like behaviour in mice. The inhibitory function of TWIST1 was mediated through the activation of the miR-214-PPAR-δ signalling pathway, resulting in mitochondrial dysfunction combined with a lack of LTP, which consequently changed dendritic cell morphology and synaptic plasticity [88]. TWIST1 knockout effectively improved depression-like behaviour such as anhedonia and failure of social interaction in CUMS mice [88]. Therefore, the maintenance of mitochondrial function not only plays an important role in providing energy for cells but also has great significance in the regulation of synaptic morphology and function.

### 2.3. MiRNA and Neurogenesis

Neurogenesis has important functions in learning, memory, and emotion regulation. Studies have shown that depression is closely associated with a reduction in hippocampal neurogenesis. The promotion of hippocampal neurogenesis effectively relieves depression-like behaviour in model animals. It has been shown that miR-139-5p is a negative regulatory factor of neural stem cell (NSC) proliferation and neuronal differentiation [91]. In the wild-type mice injected with exosomes from MDD patients, the level of miR-139-5p was significantly increased, and hippocampal neurogenesis was decreased. The intranasal injection of miR-139-5p antagonists effectively relieved depression-like behaviour (feelings of despair and approach–avoidance conflict) and prompted neurogenesis in CUMS mice [91]. In a cell model, it also confirmed that miR-139-5p antagonists can promote neurogenesis and ameliorate depression by increasing the number of doublecortin (DCX)-positive cells and new mature neurons [91]. Khandelwal et al. demonstrated that when the miRNA–mRNA network in the hippocampal DG region of CUMS mice was dysregulated, the miR-30 family (miR-30a, miR-30b, miR-30c, miR-30d, and miR-30e) was significantly downregulated, according to miRNA microarray analysis [92]. MiR-30 inhibited factors, which are required for the proliferation of NSCs/neural progenitor cells (NPCs), for example, suppressor of cytokine signalling 3 (Socs3), calcineurin subunit B type 1 (Ppp3r1), methyltransferase that methylates lysine 4 of histone H3 and activates transcription (Mll3), adhesion G protein-coupled receptor A3 (Gpr125), neuropilin-1 (Nrp-1), and runt-related transcription factor 1 (Runx1) to promote the differentiation of NSCs/NPCs in the DG region [92]. A study by Li et al. showed that miR-211-5p in the DG region of CUMS rats targeted and regulated dual specificity tyrosine-phosphorylation-regulated kinase 1A (Dyrk1A) to participate in the Dyrk1A/STAT3 signalling pathway. MiR-211-5p upregulation inhibited the Dyrk1A/STAT3 signalling pathway to promote neurogenesis in CUMS rats, reduce neuronal apoptosis, and relieve pleasure deficit, and it decreased despair and depression-like behaviour in CUMS rats [93]. MiR-34b-5p and miR-34c-5p negatively regulated Notch1 gene expression [94], whereas the Notch signalling pathway affected neuronal plasticity through the regulation of NSC proliferation and differentiation and the growth of nerve cell axons and dendrites [95]. Furthermore, studies also showed that miR-212-3p targeted the methyl-CpG-binding protein 2 (MeCP2) and downregulated its expression, resulting in the suppression of cell differentiation and proliferation, the blocking of the AKT/mTOR pathway, and the inhibition of neurogenesis [96]. Changes in the miR-17-92 level in adult hippocampal neurons significantly affected neurogenesis and anxiety and depression-related behaviour (feelings of despair) in mice. The loss of miR-17-92 reduced neurogenesis in the DG [97,98]. As a key regulator of neurogenesis, miR-19 drives the expansion of neural stem cells and radial glial cells [99]. MiR-19 has been shown to directly target the Rap guanine nucleotide exchange factor 2 (RAPGEF2) to promote cell migration and newborn neuron deposition [100]. It also inhibited phosphatase and tensin (PTEN) and promoted neural stem cell proliferation by proteins in the developing mouse cerebral cortex [99] (Table 4).

## 3. MiRNAs and Multiple Pathways Synergistically Regulate Synaptic Plasticity in Depression

### 3.1. MiRNAs and Wnt

Abnormal Wnt signalling is closely associated with numerous mental illnesses, including depression and autism. Hippocampal stem cells in adult mammals express Wnt receptors and signalling elements in the Wnt pathway. They play important roles in neurodevelopment and promote axon guidance, dendritic growth, synapse formation, and brain development. Lian et al. showed that miR-221 expression was abnormally increased in the CSF and the blood of patients with depression. Similar results were also obtained for a CUMS mouse model. In addition, miR-221 targeted and regulated Wnt2. MiR-221 knockout promoted the expression of Wnt2, p-CREB, and BDNF [101]. Therefore, miR-221 participated in the development of depression through the regulation of the Wnt2/CREB/BDNF signalling pathway [101]. Other studies also showed that miR-199a-5p and miR-383 targeted the Wnt2/CREB/BDNF signalling pathway to regulate synaptic plasticity [102,103]. Bhaskar Roy et al. showed that the abnormal upregulation of miR-128-3p was closely associated with the abnormal expression of a key gene in the Wnt signalling pathway, dishelveled-1 (DVL-1). The results of in vitro and in vivo experiments indicated that miR-128-3p targeted and negatively regulated the Dvl-1 gene. The inhibition of miR-128-3p restored Dvl-1 expression, and activated Dvl-1 activated the Wnt-β-catenin signalling pathway [104]. A study by Dai et al. indicated abnormally high miR-155 expression in the hippocampus of CUMS mice accompanied by the high expression of inflammatory cytokines TNF-a, IL-1b, and IL-6 and the low expression of DVL-1 and β-catenin. Therefore, they speculated that miR-155 participated in the regulation of depression-like behaviour (approach–avoidance conflict and anhedonia) in mice with depression through the inhibition of the Wnt-β-catenin signalling pathway, the promotion of inflammatory cytokine release, and hippocampal neuron apoptosis [105].

### 3.2. MiRNAs and CREB

cAMP responsive element binding protein (CREB) plays roles in cell proliferation, survival, and differentiation through the regulation of downstream gene expression. MiR-124 is reported to respond to 5-HT through the inhibition of CREB to enhance 5-HT-dependent long-term facilitation and regulate the synaptic plasticity of neurons. It has also been shown that miR-124 directly inhibits the Zif268 protein [106], which is very important for the stabilization of synaptic plasticity and spatial memory [107]. MiR-134 is a CNS-specific miRNA and is highly enriched in the synaptic terminals of hippocampal neurons [108]. It has been shown that SIRT1 inhibits miR-134 expression through a repressor complex containing the transcription factor Yin Yang 1 (YY1) [109]. In patients with mild cognitive impairment, loss of SIRT1 upregulated miR-134 expression, which inhibited CREB expression and reduced BDNF transcription mediated by the formation of a transcription complex containing CREB and its coactivators CREB-binding protein (CBP) and P300 [109,110], thus eventually resulting in impaired synaptic plasticity. A study by Yi et al. showed that miR-132 expression was abnormally downregulated in the hippocampus of CUMS mice. MiR-132 antagonists inhibited neuronal proliferation and postsynaptic density protein 95 (PSD95). Oleanolic acid treatment in CUMS mice for 3 weeks indicated that miR-132 upregulation was induced through the regulation of the ERK–CREB–BDNF signalling pathway to improve the learning and memory ability and synaptic plasticity of model mice [111].

### 3.3. MiRNAs and BDNF

BDNF is one of the most important and well-studied neurotrophic molecules that modify synaptic plasticity. It effectively changes synaptic strength and can be used as a mediator, regulator, or director of synaptic plasticity to participate in the development of depression. A large number of studies has shown that many miRNAs directly target BDNF to regulate its expression [112,113]. In the study of embryonic stem cells, miR-375 [114] and miR-107 [115] were found to directly target the BDNF gene. The levels of miR-375 and miR-107 were upregulated, and the level of BDNF was downregulated in ketamine-treated embryonic stem cells. The inhibition of miR-375 and miR-107 expression improved ketamine-induced neurotoxicity and nerve injury through the regulation of BDNF expression [114,115]. MiR-206, miR-30a-5p, miR-26, and miR-202-3p directly targeted BDNF to reduce the BDNF concentration [116]; therefore, they were considered to be direct inhibitors of BDNF in the prefrontal cortex [117,118]. In addition, miR-503-3p and miR-191a-5p together promoted the function of BDNF in the brain [119]. MiR-103-3p directly promoted BDNF expression and Schwann cell proliferation [120] to further decrease stress-induced hippocampal nerve injury in rats [121]. Overall, the 3′UTR of *BDNF* RNA in the human cerebral cortex can be regulated by many miRNAs.

Some regulatory functions of miRNAs on BDNF are achieved through other proteins. It has been shown that reduced MeCP2 expression delays synaptic formation [122], resulting in the dysfunction of dendrite and axon branches [123]. MiR-132 reduced BDNF expression through a reduction in the level of MeCp2 to affect the synaptic plasticity of neurons. Some miRNAs participate in the BDNF–TrkB signalling pathway [124]. Antidepressant treatment of CUMS mice revealed that changes in the levels of miR-34a-5p, miR-126, miR-200a-3p, and miR-144-3p influenced the BDNF–ERK/Akt signalling pathway and participated in the development of depression. Growing evidence showed that peripheral glial cell line-derived neurotrophic factor (GDNF) was reduced in depression. MiR-511 was found to negatively regulate GDNF family receptor alpha 1 in the basolateral amygdala in the depression, which changed the quality of GDNF signalling and then affected the downstream MAPK pathway [125] (Table 5).

**Figure 3:**
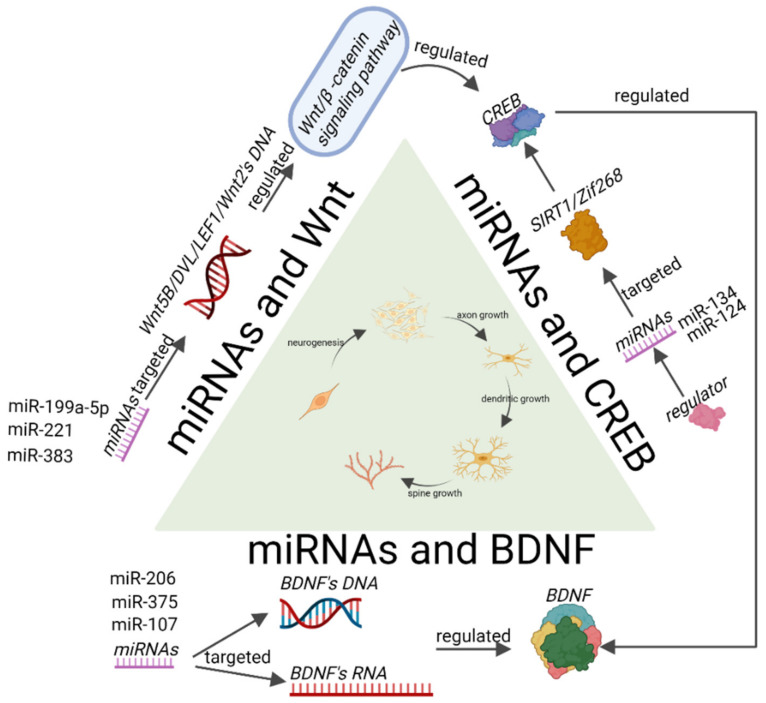
MiRNAs regulate synaptic plasticity-related signalling pathways in depression. Where miRNAs can directly target key genes in the Wnt signalling pathway and affect the expression of the Wnt signalling pathway and downstream proteins, the Wnt signalling pathway also affects the expression of CREB. MiRNAs affect the expression level and phosphorylation of CREB, which further affects downstream proteins of CREB, such as BDNF. MiRNAs affect the expression level of BDNF, and reduced BDNF expression affects synaptic plasticity. In the process of neurogenesis, axon growth, and dendrite and spine generation, miRNAs can influence each of these through the Wnt, CREB, and BDNF signalling pathways and are involved in regulating synaptic plasticity.

In summary, the influences of the Wnt signalling pathway, CREB signalling pathway, and BDNF signalling pathway on the changes in synaptic plasticity all involve miRNAs. The Wnt signalling pathway is an important adult neural regeneration pathway. It not only participates in neural regeneration through γ-aminobutyric acid (GABA) but also activates CREB to further exert its function. As one of the major upstream activation factors of BDNF, CREB is very important for the regulation of BDNF. It has been shown that the CREB/BDNF signalling pathway is of great importance in the regulation of neuronal protection and regeneration, in formation of synapses, and in improvements in learning and memory. Therefore, we believe that the activation of the CREB signalling pathway by the Wnt signalling pathway indirectly increases the BDNF concentration. These three pathways work together to influence BDNF expression to further affect synaptic plasticity. In addition, miRNAs play a major role in this process (Figure 3).

## 4. MiRNAs and the Diagnosis and Treatment of Depression

Changes in the levels of miRNAs in the peripheral blood of patients with depression are important indicators in the diagnosis and treatment of depression. For example, high throughput analysis results indicated changes in the expression levels of has-miR-107, miR-133a, miR-148a, miR-200c, miR-381, miR-425-3p, miR-494, miR-517b, miR-579, miR-589, miR-636, miR-652, miR-941, and miR-1243 of which significantly high miR-941 and miR-589 expression was closely associated with disease severity. Bioinformatics prediction results indicated that miR-941 and miR-589 were closely associated with PPT1, TNF, IL1B, and HIST1H1E [126]. This result suggested that changes in the levels of miRNAs that were closely associated with the disease course could be used as candidate biomarkers for diagnosing depression. Furthermore, some studies also showed that miRNAs might be involved in the mechanism of action of antidepressants. For example, after duloxetine treatment in patients with major depressive disorder and CUMS mice, the miR-146a-5p, miR-24-3p, and miR-425-3p expression in blood was downregulated. Target prediction and pathway analysis results indicated that they were closely associated with the MAPK/Wnt signalling pathway. After duloxetine treatment in human neural progenitor cells, miR-146b-5p, miR-24-3p, and miR-425-3p were significantly downregulated, and 92% of the MAPK/Wnt pathway-related genes in cells exhibited changes in expression. These results indicated that there were interactions among the four miRNAs, MAPK/Wnt signalling pathway, and antidepressant treatment. These four miRNAs could be used as blood markers during antidepressant treatment [127]. A study by Li et al. showed that miR-207 in exosomes entered into astrocytes through the blood–brain barrier to target TLR4-interactor with leucine-rich repeats (Tril) and inhibit NF-κB signalling, thus reducing inflammatory cytokine release and relieving stress symptoms in mice [128]. It has also been shown that isoliquiritin significantly ameliorates depressive symptoms in CUMS mice. Cell experiment results indicated that isoliquiritin protected primary microglia from LPS- and ATP-elicited NLRP3 inflammasome activation. The presentations were a reduction in the protein levels of p-NF-κB, NLRP3, cleaved caspase-1, IL-1β, and GSDMD-N and the promotion of miR-27a expression. MiR-27a inhibitors reversed isoliquiritin-generated antidepression effects [129]. Thus, these results indicated that miRNAs level played a very important role in improving the symptoms of patients with depression. In addition, miRNAs hold great potential as candidate biomarkers for diagnosis and therapy for other diseases. Therefore, miRNAs in peripheral tissues, especially in blood, can be used as biomarkers or potential drug targets for the diagnosis or treatment of depression.

## 5. Discussion and Outlook

Depression has become an important scientific problem in neuroscience and psychology. Currently, depression is mainly diagnosed by professional physicians by using the Hamilton Depression Rating Scale (HAMD) followed by a routine examination. This diagnostic method relies on the subjectivity of doctors and patients to some extent. Therefore, it is desirable to find effective and objective methods for diagnosing depression. In addition, identifying novel biomarkers has important significance in the diagnosis of depression.

A large amount of evidence indicates that miRNAs in neurons are involved in the regulation of protein translation in various parts of the cell body and in synapses. They target multiple synaptic proteins and translation regulatory factors to regulate synaptic plasticity. Therefore, the development of compounds that target miRNAs or miRNA-regulated genes might be an effective method for the treatment of depression. Changes in miRNA levels in peripheral tissues reflect their levels in the brain. In addition, miRNAs can penetrate the blood–brain barrier and enter into the central system through exosomes as carriers to negatively regulate many physiological processes in the CNS; for example, they regulate neurotransmitter transmission, synaptic energy metabolism, synaptic morphology and structure, and neurogenesis. Therefore, the expression levels of miRNAs in peripheral tissue can be used not only as a method for diagnosing depression but also as targets for treating depression. The most prominent evidence for supporting miRNAs as targets for treating depression is that drugs currently used for treating depression change the expression of some miRNAs. In addition, directly targeting miRNAs may be a safer and more effective route and may produce faster treatment effects and fewer side effects.

However, these studies have certain limitations. The first is the heterogeneity of depression. For example, different depressed patients have different expression levels of pathogenesis-related miRNAs due to the differences in physical conditions, etiologies, and living environments. Zhang et al. studied miRNA expression levels in plasma and brain tissues of patients with depression and reported that miR-134 was significantly downregulated in plasma and CSF [101] However, a study by Gao et al. showed that miR-134 expression in the prefrontal cortex was upregulated. The second is that depression is associated with genetic susceptibility. As mentioned above, some miRNAs in patients with depression involve single nucleotide polymorphisms (SNPs) and genetic variations, for example, SERT gene-associated miR-16 and SIRT1 gene-associated miR-134. These findings need to be confirmed by further studies with an adequate sample size. Therefore, studying whether miRNAs affect synaptic structure and function and whether these miRNAs regulate synaptic proteins that are involved in the pathogenesis of depression is very important.

## Figures and Tables

**Table 1:** MiRNAs involved in neurotransmitter transmission in depression.

MicroRNAs	Study Model	Source	Target	The Types of Neurotransmitters	References
miR-16	Human Rat	Hippocampus, CSF	SERT	DA	[27,28]
miR-384-5p	Rat	Prefrontal cortex	DRD1	DA	[32]
miR-504	Rat	Nucleus accumbens	DRD1	DA	[33]
miR-125b	Mouse	Hippocampus	NR2A	Glu	[35]
miR-223	Mouse	Hippocampus	GluN2B GluA2	Glu	[44,45]
miR-92a	Rat	Hippocampus	GluA1	Glu	[39]
miR-137	Rat	Cortex	Grin2A	Glu	[41]
miR-320a	Human	Plasma	GRIN2A	Glu	[36]
miR-501-3p	Rat	Hippocampus	GluA1	Glu	[42,43]
miR-335	Human	Blood	GRM4	Glu	[50,51]
miR-1202	Human	Cortex, Blood	GRM4	Glu	[52,53]
miR-124a	Mouse	Embryonic stem cells,	GLT1	Glu	[55]

**Table 2:** MiRNAs involved in synaptic vesicle transport in depression.

MicroRNAs	Study Model	Source	Target	References
miR-451a	Human	Plasma	SLC17A7	[34]
miR-15b-5p	Mouse	Nucleus accumbens	STXBP3A/VAMP1	[55]
miR-485	Mouse	Hippocampus	SV2A	[34]
miR-133a	Human	Amygdala	SV2A	[56]
miR-128	Human	Amygdala	SV2A	[56]
miR-96	Mouse	Hippocampus	SV2C	[57]
miR-34a-5p	Mouse	Hippocampus	SYT1	[57]

**Table 3:** MiRNAs involved in synaptic structural plasticity in depression.

MicroRNAs	Study Model	Source	Target	References
miR-132	Mouse	Hippocampus	p250GAP	[60,61]
miR-138	Mouse	Cortex, Hippocampus, The cerebellum	SIRT1	[62]
miR-138	Rat	Hippocampus	APT1	[62]
miR-9	Mouse		REST	[63,64]
miR-137	Human, Mouse	Cortex	Ezh2	[65]
miR-188	Mouse	Hippocampus	Nrp-2	[66]
miR-485	Mouse	Hippocampus	SNCA, MAP, GRIN1	[67]
miR-497	Rat	Hippocampus	FGF2	[72,73]
miR-185	Human, Mouse		TrkB-T1	[70]
miR-192-5p	Mouse	Hippocampus	Fbln2	[71]
miR-191	Mouse	Hippocampus	Tmod2	[72]
miR-298-5p	Mouse	Hippocampus	NOX1	[76]
miR-210	Mouse	Hippocampal neurons	Hif-1α	[77]
miR-99a	Mouse	Hypothalamus	FKBP51	[73]
miR-214	Mouse	Cortex, Hippocampus	PPAR-δ	[82,84]

**Table 4:** MiRNAs involved in neurogenesis in depression.

MicroRNAs	Study Model	Source	Target	References
miR-139-5p	Mouse	Hippocampus, Blood		[85]
miR-34b/c-5p	Human	Hippocampus, Blood	NOTCH1	[88]
miR-212-5p	Rat	Hippocampus	MeCP2	[90]
miR-17-92	Mouse	Hippocampus	Sgk1	[91]
miR-124	Rat	Hippocampus	CREB	[92,93]

**Table 5:** MiRNAs involved in neurotransmitter transmission in depression.

MicroRNAs	Study Model	Source	Target	References
miR-200a-3p	Mouse	Hippocampus	APT1	[62]
miR-124	Rat	Hippocampus	CREB	[93]
miR-221	Human, Mouse	Blood, CSF, Hippocampus	Wnt	[94]
miR-128-3p	Rat	Amygdala	Wnt	[97]
miR-155	Mouse	Hippocampus	Wnt	[98]
miR-134	Human	Hippocampus	BDNF	[100,101,102]
miR-134	Human		CREB	[101,102]
miR-132	Mouse	Hippocampus	CREB	[103]
miR-375	Human, Mouse	Embryonic stem cells	BNDF	[106,107]
miR-107	Rat	Embryonic stem cells	BNDF	[106,107]
miR-206	Human	Nerve cells	BNDF	[108]
miR-30a-5p		Prefrontal cortex	BNDF	[109,110]
miR-26		Prefrontal cortex	BNDF	[109,110]
miR-503-3p	Rat	Hippocampus	BNDF	[111]
miR-191a-5p	Rat	Hippocampus	BNDF	[111]
miR-103-3p	Rat	Hippocampus	BDNF	[112]
miR-202-3p	Rat	Hippocampus	BDNF	[113]
miR-199a-5p	Human, Mouse	Blood, CSF, Hippocampus	Wnt	[113]
miR-383	Human, Mouse	Blood, CSF, Hippocampus	Wnt	[114]
miR-132	Human		BDNF	[114,115]
miR-124	Rat	Hippocampus	BDNF	[117]
miR-126	Mouse		BDNF	[118]
miR-34a-5p	Mouse		BDNF	[118,119]
miR-144-3p	Mouse		BDNF	[118,120]

## Data Availability

Not applicable.
